# Hepatic Surgery

**Published:** 1890-07-12

**Authors:** 


### HEPATIC SURGERY.

Ponfick, of Breslau, gave at the recent surgical congress at Berlin an account of experiments performed by him on the extirpation of the livers of rabbits, with a view of estimating the feasibility of such a procedure in the human subject in cases of disease of that organ. He was astonished at finding that after $\frac{3}{4}$ or even $\frac{5}{6}$ of the liver had been removed regeneration commenced, often within a few days, the organ in time resuming its original dimensions, or even becoming larger than before. But Dr. Julius Hochenegg, assistant surgeon at Professor Albert's clinic at Vienna, had already successfully performed the operation without premeditation. While removing a diseased gall bladder he found that the overlying portion of the liver was infiltrated with cancerous growth. Getting an assistant to grasp the lobe firmly, he excised the whole of the diseased portion together with a sufficient margin of sound tissue. The hæmorrhage was not excessive, but failing in his attempts to bring together the margins of the hepatic surface, from the sutures cutting their way through the fragile tissue, he laid pads of iodoform gauze on each side of the abdominal section, and carrying deep sutures, "mattrass stitches," through the skin, muscles and liver secured the cut surface of the latter in the wound, which he dressed with iodoform and sublimate gauze. The subsequent progress of recovery was rapid and uninterrupted, the margins of the parietal and visceral peritoneum united, and the wound healed by granulation, leaving the liver of course adherent to the anterior wall of the abdomen. Having by his extemporised procedure obviated the one greatly-feared danger of internal hæmorrhage, and the operation having been completely successful, he strongly urged further trials in suitable cases, those, namely, where cancer was confined to the margin or a limited portion of the organ.

